# Serum JKAP as a potential prognostic biomarker in acute coronary syndrome patients undergoing percutaneous coronary intervention

**DOI:** 10.3389/fcvm.2025.1631896

**Published:** 2025-12-12

**Authors:** Xinjing Chen, Jingxuan Hong

**Affiliations:** Department of Cardiology, Provincial Clinical Medical College of Fujian Medical University, Fuzhou University Affiliated Provincial Hospital, Fuzhou, Fujian, China

**Keywords:** acute coronary syndrome, percutaneous coronary intervention, JKAP, CD4^+^ T cells, prognosis

## Abstract

**Objective:**

Our previous studies revealed that knockdown of JNK pathway-associated phosphatase (JKAP) facilitates atherosclerotic progression by regulating CD4^+^ T-cell differentiation. Moreover, CD4^+^ T-cell subtypes are dysregulated and can serve as a prognosis biomarker in acute coronary syndrome (ACS) patients who have undergone percutaneous coronary intervention (PCI). This study aims to further investigate the correlation between JKAP with CD4^+^ T-cell subtypes and prognosis in ACS patients who have undergone PCI.

**Methods:**

This study included 173 ACS patients, for whom serum JKAP levels were detected before PCI and 1 month (M1) after PCI via an enzyme-linked immunosorbent assay. Short physical performance battery (SPPB) scores at M1 and major adverse cardiac event (MACE) data were collected. Moreover, serum JKAP levels were detected in 32 healthy control subjects.

**Results:**

JKAP levels were significantly lower in ACS patients vs. healthy control subjects (*P* < 0.001) and were increased at M1 after PCI vs. before PCI in ACS patients (*P* < 0.001). JKAP negatively correlated with T-helper (Th)17 cells (*P* = 0.002) and tended to negatively associate with Th1 cells (*P* = 0.060), but did not correlate with Th2 or T-regulatory cells. Importantly, JKAP before PCI and at M1 after PCI showed good values in predicting MACE risk via receiver operating curve analyses [area under curve (AUC) = 0.721, AUC = 0.778, respectively]; however, JKAP demonstrated relatively weaker values in predicting an SPPB score of ≤6 points at M1 (AUC = 0.638, AUC = 0.649, respectively). Multivariable analyses revealed that JKAP at M1 after PCI independently predicted a lower risk of MACE (*P* = 0.045) and an SPPB score of ≤6 points at M1 (*P* = 0.025).

**Conclusion:**

JKAP may serve as a prognostic biomarker in ACS patients who have undergone PCI.

## 1 Introduction

Acute coronary syndrome (ACS) is one of the leading causes of global morbidity and mortality, encompassing ST-segment elevation myocardial infarction (STEMI), non-ST-segment elevation myocardial infarction (NSTEMI), and unstable angina (UA) (1). The underlying pathogenesis of ACS primarily stems from the destabilization of coronary atherosclerotic plaques, and vulnerable plaques—characterized by thin fibrous caps, large lipid cores, and inflammatory cell infiltration—may rupture or erode, triggering platelet aggregation and thrombus formation, ultimately leading to partial or complete vascular occlusion (2, 3). Percutaneous coronary intervention (PCI) serves as a cornerstone in the management of ACS (4). In cases of STEMI, early PCI facilitates rapid reperfusion of the occluded vessel, salvaging ischemic myocardium, significantly reducing mortality, and improving long-term outcomes; in cases of NSTEMI and UA, PCI aids in plaque stabilization and mitigates the risk of recurrent ischemia (4–8).

JNK pathway-associated phosphatase (JKAP), also called dual-specificity phosphatase 22 (DUSP22), can dephosphorylate the phosphorylated residues of tyrosine, serine, and threonine (9, 10). JKAP is reported to specifically regulate the JNK pathway and modify ERK and NF-κB pathways via dephosphorylating focal adhesion kinase (11, 12); the three pathways are critical for the signaling involved in immunity and inflammation responses (13–15). Moreover, JKAP represses the TCR signaling-mediated T-cell activation, Lck-mediated immune response and autoimmunity, and the secretion of inflammatory cytokines (9, 10, 16–18). Immunity and inflammation are two important triggers for the development and progression of ACS (19–21). Based on these findings, we hypothesized that JKAP might be related to the risk and prognosis of ACS.

We have conducted two previous studies on related topics (22, 23). In the first study, we observed that the deficient JKAP levels promoted atherosclerotic progression by regulating CD4^+^ T-cell differentiation and inflammatory cytokines through ERK and NF-κB pathways (22). In the other study, we discovered that blood CD4^+^ T-cell subtypes including T helper (Th) 1, Th2, Th17, and T regulatory (Treg) cells were dysregulated in ACS patients and changed during PCI treatment; moreover, Th1 and Th17 cell proportions could predict physical function recovery and major adverse cardiac event (MACE) risk in these patients (23).

Therefore, in this study, we aim to further investigate the abnormal level of serum JKAP, its correlation with CD4^+^ T-cell subtypes and clinical characteristics, and its prognostic value in ACS patients who have undergone PCI.

## 2 Methods

### 2.1 Participants

A total of 173 ACS patients who underwent a PCI procedure between February 2020 and November 2021 were included in this study. The main eligibility criteria were ACS diagnosis, age above 18 years, having undergone a PCI procedure, survival during hospitalization, and normal discharge. Detailed inclusion and exclusion criteria of ACS patients can be found in our previous study (23). Moreover, 32 healthy control subjects were also included, with the inclusion and exclusion criteria as established in our previous study (23). This study was approved by the Ethics Committee of Fuzhou University Affiliated Provincial Hospital (approval number K2024-09-014). Informed consent was obtained from the participants or their immediate families.

### 2.2 Data collection and prognosis information

This following information relating to ACS patient characteristics was collected for this study: demographics, disease history, biochemical indexes, and disease-related and PCI-related information. The short physical performance battery (SPPB) score was evaluated 1 month (M1) following PCI. The scoring criteria for SPPB were adopted from a previous study (24), and an SPPB score of ≤6 was defined as a mild limitation of physical performance, based on criteria established in our previous study (23). MACE was evaluated according to the follow-up data. The criteria for MACE were established in another study (25), which documented the occurrence of all-cause mortality, acute myocardial infarction, or unplanned coronary revascularization, whichever occurred first.

### 2.3 Detections

Serum JKAP levels in ACS patients were detected before PCI and at M1 after PCI using an enzyme-linked immunosorbent assay commercial kit (Meilian Bio-tech, Shanghai, China), in accordance with the manufacturer’s instructions. Th1, Th2, Th17, and Treg cell proportions in CD4^+^ T cells of peripheral blood mononuclear cells (PBMCs) from ACS patients were measured before PCI through the application of the Th1/Th2/Th17 Staining Kit (Multi Science, Hangzhou, China) and Regulatory T-Cell Staining Kit (Multi Science, Hangzhou, China) using a flow cytometry assay. The detailed information on flow cytometry has been provided in our previous study (23). Moreover, serum JKAP levels from healthy control subjects were also measured after the inclusion.

### 2.4 Statistics

SPSS version 26.0 (IBM, Armonk, USA) and GraphPad version 9.0 (GraphPad, San Diego, USA) were used for statistical analyses and graphs, respectively. Comparisons were determined using the Wilcoxon rank-sum test, Wilcoxon signed-rank test, or Kruskal–Wallis test, as appropriate. Correlations were determined using Spearman's rank correlation test. The ability of the parameters to distinguish patients with an SPPB score ≤ 6 at M1 from those with an SPPB score > 6 at M1 was determined by a receiver operating characteristic (ROC) curve and the presence of area under the curve (AUC). The same approach was used to evaluate the ability of the parameters to distinguish patients who experienced MACE from those who did not. Cumulative MACE risk was determined using the Kaplan–Meier curve. Furthermore, all biochemical parameters were included in the analyses of independent parameters related to risk of an SPPB score of ≤6 at M1 or MACE using multivariable logistic regression with the conditional forward method that utilized the following variables: JKAP, Th1, Th2, Th17, Treg, red blood cell (RBC), hemoglobin (Hb), white blood cell (WBC), alanine transaminase (ALT), aspartate transaminase (AST), serum creatinine (Scr), blood glucose, triglycerides (TG), total cholesterol (TC), low-density lipoprotein cholesterol (LDL-C), high-density lipoprotein cholesterol (HDL-C), high-sensitive C-reactive protein (hsCRP), cardiac troponin I (cTnI), and creatine kinase-MB (CK-MB). A *P* < 0.05 was considered to be statistically significant.

## 3 Results

### 3.1 Characteristics of ACS patients

A total of 173 ACS patients were included at the age range of 61.2 ± 7.8 years, comprising 24.9% females and 75.1% males. Of these, 69.9%, 45.1%, and 30.1% of the patients had a medical history of hypertension, hyperlipidemia, and diabetes, respectively. The majority of the patients were diagnosed with STEMI (64.2%), while 17.3% of the patients had NSTEMI and 18.5% had UA. The symptom-to-balloon time was 5.0 [interquartile range (IQR): 3.0–13.5] h. The clinical characteristics of the ACS patients are presented in Table 1.

**Table 1:** Clinical characteristics of ACS patients.

Items	ACS patients (*N* = 173)
Demographics	
Age, mean ± SD (years)	61.2 ± 7.8
Female [*n* (%)]	43 (24.9)
BMI, mean ± SD (kg/m^2^)	25.5 ± 3.4
Current smoker [*n* (%)]	65 (37.6)
Disease history	
Hypertension [*n* (%)]	121 (69.9)
Hyperlipidemia [*n* (%)]	78 (45.1)
Diabetes [*n* (%)]	52 (30.1)
Previous PCI [*n* (%)]	0 (0.0)
Previous CABG [*n* (%)]	0 (0.0)
Family history of CAD [*n* (%)]	49 (28.3)
Blood biochemical indexes	
RBC, median (IQR) (10^12^/L)	4.7 (4.1–5.1)
Hb, median (IQR) (g/L)	139.0 (122.5–154.0)
WBC, median (IQR) (10^9^/L)	8.9 (6.8–11.1)
ALT, median (IQR) (U/L)	24.5 (15.2–35.8)
AST, median (IQR) (U/L)	27.8 (18.1–40.4)
Scr, median (IQR) (μmol/L)	77.6 (66.2–88.1)
Blood glucose, median (IQR) (mmol/L)	6.8 (5.8–8.3)
TG, median (IQR) (mmol/L)	1.8 (1.1–2.6)
TC, median (IQR) (mmol/L)	5.0 (4.2–5.8)
LDL-C, median (IQR) (mmol/L)	3.1 (2.4–3.8)
HDL-C, median (IQR) (mmol/L)	1.0 (0.8–1.2)
hsCRP, median (IQR) (mg/L)	2.7 (1.8–4.1)
cTnI, median (IQR) (ng/mL)	2.3 (0.5–3.3)
CK-MB, median (IQR) (ng/mL)	22.2 (8.4–45.6)
Disease-related characteristics	
Manifestation [*n* (%)]	
STEMI	111 (64.2)
NSTEMI	30 (17.3)
UA	32 (18.5)
Symptom-to-balloon time, median (IQR) (h)	5.0 (3.0–13.5)
Target lesion at LAD [*n* (%)]	99 (57.2)
Target lesion at LCX [*n* (%)]	43 (24.9)
Target lesion at RCA [*n* (%)]	74 (42.8)
Multiple target lesions [*n* (%)]	43 (24.9)
PCI-related characteristics	
PCI type [*n* (%)]	
DCB	21 (12.1)
DES	152 (87.9)
DCB or DES diameter, median (IQR) (mm)	3.0 (2.8–3.5)
DCB or DES length, median (IQR) (mm)	33.0 (23.0–41.0)

ACS, acute coronary syndrome; SD, standard deviation; BMI, body mass index; PCI, percutaneous coronary intervention; CABG, coronary artery bypass grafting; CAD, coronary artery diseases; RBC, red blood cell; IQR, interquartile range; Hb, hemoglobin; WBC, white blood cell; ALT, alanine transaminase; AST, aspartate transaminase; Scr, serum creatinine; TG, triglycerides; TC, total cholesterol; LDL-C, low-density lipoprotein cholesterol; HDL-C, high-density lipoprotein cholesterol; hsCRP, high-sensitive C-reactive protein; cTnI, cardiac troponin I; CK-MB, creatine kinase-MB; STEMI, ST-segment elevation myocardial infarction; NSTEMI, non-ST-segment elevation myocardial infarction; UA, unstable angina; LAD, left anterior descending artery; LCX, left circumflex artery; RCA, right coronary artery; DCB, drug-coated balloon; DES, drug-eluting stent.

### 3.2 JKAP level in ACS patients

**Figure 1:**
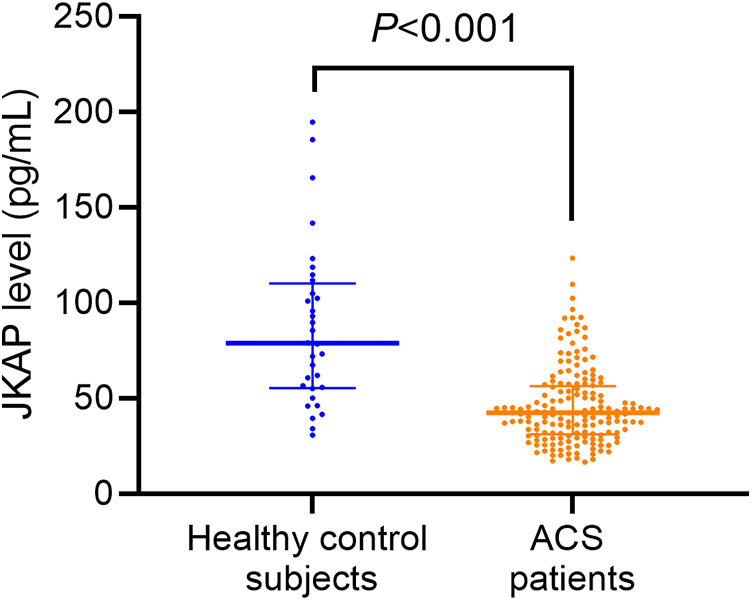
JKAP levels between ACS patients and healthy control subjects.

**Figure 2:**
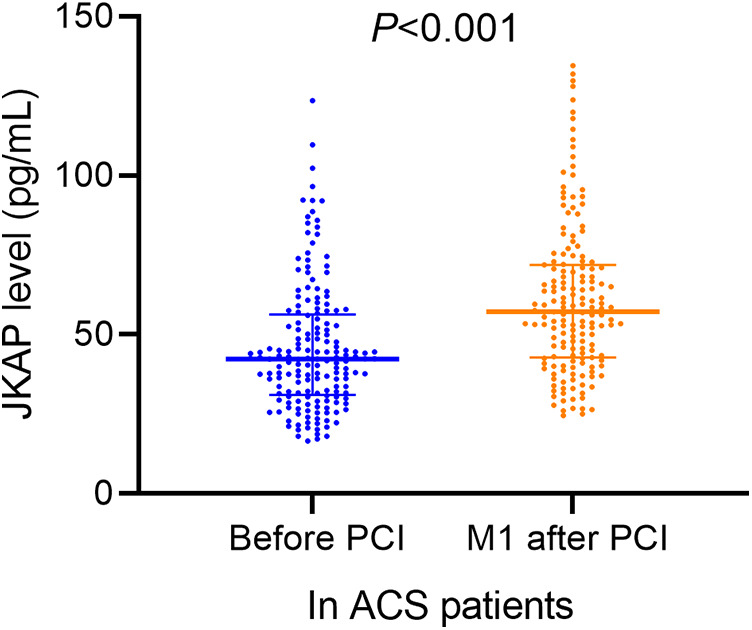
JKAP levels before PCI and at M1 after PCI.

Serum JKAP levels were predominantly lower in ACS patients vs. healthy control subjects [median (IQR): 42.3 (31.1–56.4) vs. 78.8 (55.4–110.1) pg/mL, *P* < 0.001, Figure 1]. Moreover, serum JKAP levels were increased from 42.3 (31.1–56.4) pg/mL before PCI to 57.2 (IQR: 42.8–71.9) pg/mL at M1 after PCI in ACS patients (*P* < 0.001, Figure 2).

### 3.3 Correlation between JKAP level and CD4^+^ cell subtypes in ACS patients

Serum JKAP levels were negatively correlated with Th17 cells (*r* = −0.235, *P* = 0.002), and showed a tendency to be reversely associated with Th1 cells, but did not reach statistical significance (*r* = −0.143, *P* = 0.060) in ACS patients (Table 2). However, JKAP levels were not correlated with Th2 cells (*r* = 0.119, *P* = 0.120) or Treg cells (*r* = 0.092, *P* = 0.231) in ACS patients.

**Table 2:** Correlation between JKAP and CD4^+^ T cells in ACS patients.

Items	JKAP level before PCI	
	*r* Correlation coefficient	*P*-value
Th1 cells before PCI	−0.143	0.060
Th2 cells before PCI	0.119	0.120
Th17 cells before PCI	−0.235	0.002
Treg cells before PCI	0.092	0.231

JKAP, JNK pathway-associated phosphatase; ACS, acute coronary syndrome; PCI, percutaneous coronary intervention; Th, T helper; Treg, T regulatory.

### 3.4 Correlation between JKAP levels and characteristics in ACS patients

In terms of continuous clinical characteristics, serum JKAP levels were negatively correlated with blood glucose (*r* = −0.166, *P* = 0.029), hsCRP (*r* = −0.261, *P* = 0.001), and cTnI (*r* = −0.152, *P* = 0.046) in ACS patients (Table 3). Regarding categorical clinical characteristics, JKAP levels were negatively associated with the medical history of hyperlipidemia (*P* = 0.043) and medical history of diabetes (*P* < 0.001) in ACS patients (Table 4). However, no correlation was found in ACS patients between JKAP levels and other clinical characteristics.

**Table 3:** Correlation between JKAP and continuous clinical characteristics in ACS patients.

Items	JKAP level before PCI	
	*r* Correlation coefficient	*P*-value
Age (years)	−0.082	0.281
BMI (kg/m^2^)	−0.118	0.123
RBC (10^12^/L)	−0.073	0.341
Hb (g/L)	−0.112	0.142
WBC (10^9^/L)	−0.025	0.742
ALT (U/L)	−0.148	0.053
AST (U/L)	−0.085	0.268
Scr (*μ*mol/L)	−0.072	0.344
Blood glucose (mmol/L)	−0.166	0.029
TG (mmol/L)	0.024	0.755
TC (mmol/L)	0.015	0.847
LDL-C (mmol/L)	−0.005	0.952
HDL-C (mmol/L)	0.105	0.169
hsCRP (mg/L)	−0.261	0.001
cTnI (ng/mL)	−0.152	0.046
CK-MB (ng/mL)	−0.133	0.082
Symptom-to-balloon time (h)	−0.059	0.444
DCB or DES diameter (mm)	−0.008	0.917
DCB or DES length (mm)	−0.065	0.393

JKAP, JNK pathway-associated phosphatase; ACS, acute coronary syndrome; PCI, percutaneous coronary intervention; BMI, body mass index; RBC, red blood cell; Hb, hemoglobin; WBC, white blood cell; ALT, alanine transaminase; AST, aspartate transaminase; Scr, serum creatinine; TG, triglycerides; TC, total cholesterol; LDL-C, low-density lipoprotein cholesterol; HDL-C, high-density lipoprotein cholesterol; hsCRP, high-sensitive C-reactive protein; cTnI, cardiac troponin I; CK-MB, creatine kinase-MB; DCB, drug-coated balloon; DES, drug-eluting stent.

**Table 4:** Correlation between JKAP and categorical clinical characteristics in ACS patients.

Items	JKAP level before PCI	
	Median (IQR) (pg/mL)	*P*-value
Gender		0.975
Male	42.4 (30.8–56.8)	
Female	42.3 (32.4–53.4)	
Current smoker		0.995
No	42.0 (31.0–57.5)	
Yes	42.8 (31.1–52.7)	
Hypertension		0.800
No	42.0 (31.8–56.3)	
Yes	42.4 (30.6–56.6)	
Hyperlipidemia		0.043
No	42.8 (32.4–58.2)	
Yes	39.0 (28.4–51.0)	
Diabetes		<0.001
No	43.1 (35.6–60.4)	
Yes	35.9 (24.4–45.1)	
Family history of CAD		0.379
No	42.5 (32.0–57.0)	
Yes	41.0 (28.9–54.3)	
Manifestation		0.332
STEMI	40.7 (30.6–53.4)	
NSTEMI	46.2 (32.2–67.9)	
UA	42.3 (29.6–55.2)	
Target lesion at LAD		0.784
No	41.8 (31.7–53.5)	
Yes	42.4 (30.4–57.5)	
Target lesion at LCX		0.509
No	42.4 (31.5–57.2)	
Yes	41.3 (30.4–52.8)	
Target lesion at RCA		0.516
No	42.4 (30.4–57.6)	
Yes	41.4 (31.5–52.2)	
Multiple target lesions		0.276
No	42.4 (31.7–57.4)	
Yes	40.4 (28.7–50.2)	
PCI type		0.496
DCB	39.3 (27.3–53.3)	
DES	42.4 (31.4–57.0)	

JKAP, JNK pathway-associated phosphatase; ACS, acute coronary syndrome; PCI, percutaneous coronary intervention; IQR, interquartile range; CAD, coronary artery diseases; STEMI, ST-segment elevation myocardial infarction; NSTEMI, non-ST-segment elevation myocardial infarction; UA, unstable angina; LAD, left anterior descending artery; LCX, left circumflex artery; RCA, right coronary artery; DCB, drug-coated balloon; DES, drug-eluting stent.

### 3.5 Prognostic value of JKAP to PCI treatment in ACS patients

The SPPB score at M1 and MACE information after the PCI procedure were collected for prognostic evaluation in ACS patients. Subsequently, the SPPB score at M1 after PCI was found to be 8.0 ± 1.5 points (Supplementary Figure S1A), with 20.7% patients experiencing a mild limitation in physical performance that was defined as an SPPB score of ≤6 points (Supplementary Figure S1B). Regarding MACE, using the Kaplan–Meier curve, the 1-year accumulated MACE risk was 6.6% ± 2.3% and the 2-year accumulated MACE risk was 15.2% ± 4.4% (Supplementary Figure S1C). According to direct calculations, 7.5% of patients experienced MACE while the other 92.5% of patients did not (Supplementary Figure S1D).

**Figure 3:**
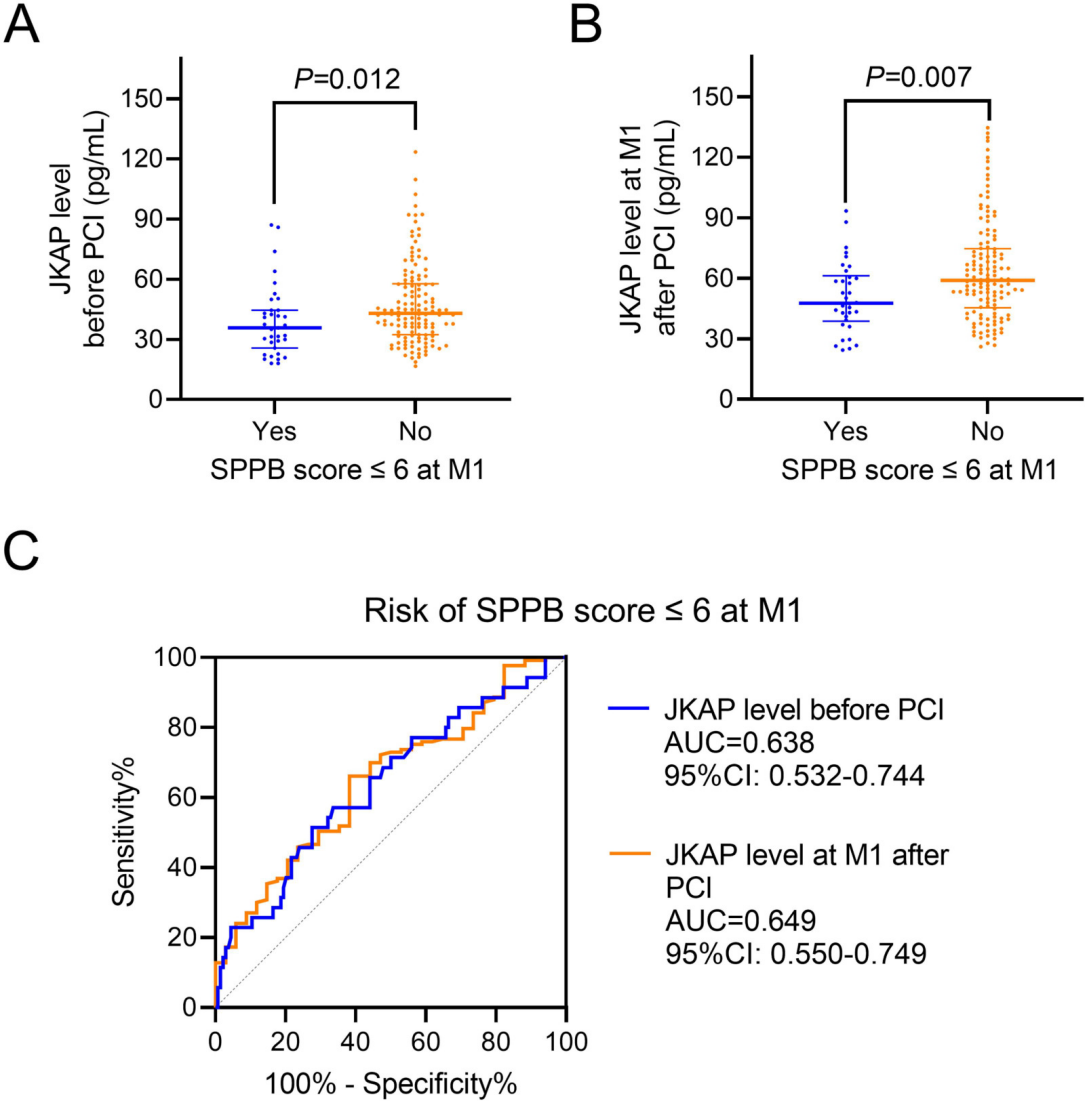
Correlation between JKAP levels and physical function recovery. Comparison of JKAP levels before PCI **(A)** and at M1 after PCI **(B)** between patients having an SPPB score ≤6 at M1 and patients having an SPPB score ≥6. ROC analyses of JKAP levels before PCI and at M1 after PCI for the risk of SPPB score ≤6 at M1 **(C)**.

Serum JKAP levels before PCI were lower in patients with an SPPB score of ≤6 at M1 vs. those with scores ≥6 [median (IQR): 35.8 (25.7–44.6) vs. 43.0 (32.4–57.7) pg/mL, *P* = 0.012, Figure 3A]. JKAP levels at M1 after PCI were also lower in patients with an SPPB score of ≤6 at M1 vs. those with scores ≥6 [median (IQR): 47.7 (38.8–61.3) vs. 59.0 (45.4–74.7) pg/mL, *P* = 0.007, Figure 3B]. Moreover, JKAP levels before PCI (AUC = 0.638, 95% CI: 0.532–0.744) and JKAP at M1 after PCI (AUC = 0.649, 95% CI: 0.550–0.749) could predict the risk of an SPPB score of ≤6 at M1 in ACS patients who had undergone PCI (Figure 3C), but the predictive values were not strong (both AUC < 0.700).

**Figure 4:**
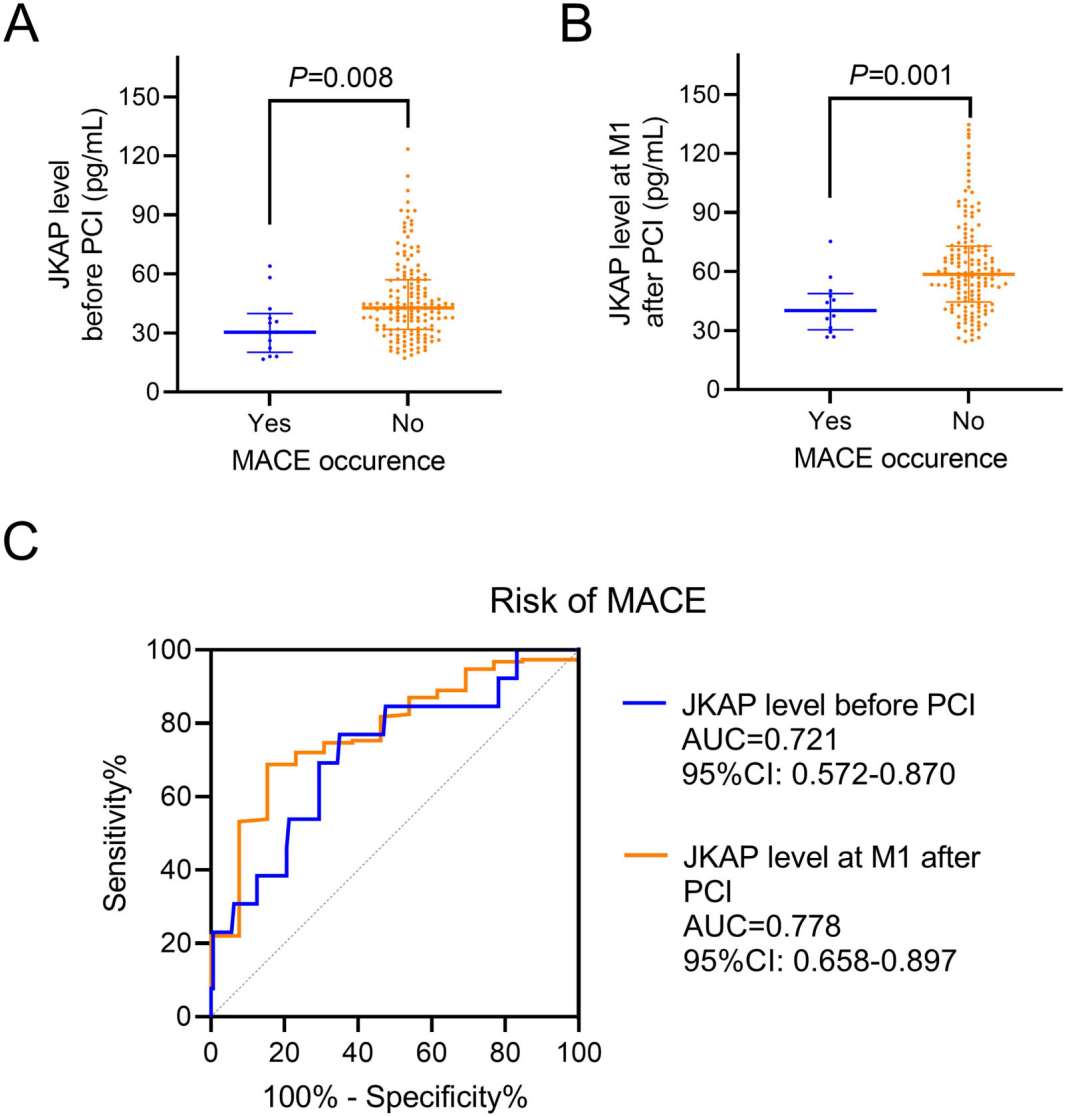
Correlation between JKAP levels and MACE. Comparison of JKAP levels before PCI **(A)** and at M1 after PCI **(B)** between patients who experienced MACE and patients who did not. ROC analyses of JKAP levels before PCI and at M1 after PCI for the risk of MACE **(C)**.

Significantly, JKAP levels before PCI were reduced in patients who experienced MACE vs. those who did not [median (IQR): 30.4 (20.2–39.9) vs. 42.7 (31.8–57.0) pg/mL, *P* = 0.008, Figure 4A]. JKAP levels at M1 after PCI were also lower in patients who experienced MACE vs. those who did not [median (IQR): 40.2 (30.4–48.9) vs. 58.6 (44.6–72.8) pg/mL, *P* = 0.001, Figure 4B]. Furthermore, serum JKAP levels before PCI (AUC = 0.721, 95% CI: 0.572–0.870) and JKAP at M1 after PCI (AUC = 0.778, 95% CI: 0.658–0.897) were able to predict risk of MACE in ACS patients who had undergone PCI (Figure 4C), and the predictive values were robust (both AUC > 0.700).

### 3.6 Prognostic value of JKAP and other biochemical parameters in PCI treatment among ACS patients

All biochemical parameters were included in the multivariable logistic regression with conditional forward method, to analyze their independent correlation with the risk of SPPB score ≤6 at M1 and MACE, respectively (Table 5). JKAP levels at M1 after PCI [odds ratio (OR) = 0.976, *P* = 0.025] were independently correlated with a lower risk of an SPPB score of ≤6 at M1, while Scr (OR = 1.021, *P* = 0.044) and cTnI (OR = 1.186, *P* = 0.018) were independently associated with a higher risk of an SPPB score of ≤6 at M1. Regarding MACE risk, JKAP levels at M1 after PCI (OR = 0.950, *P* = 0.045) and Treg cells (OR = 0.523, *P* = 0.043) could independently predict a lower risk of MACE, but Scr (OR = 1.036, *P* = 0.016) and hsCRP (OR = 1.317, *P* = 0.026) could independently predict a higher risk of MACE.

**Table 5:** Multivariable logistic regression analyses with conditional forward method.

Parameters	*P*-value	OR	95% CI
Risk of SPPB score ≤6 at M1			
JKAP level at M1 after PCI (pg/mL)	0.025	0.976	0.956–0.997
Scr (μmol/L)	0.044	1.021	1.001–1.041
cTnI (ng/mL)	0.018	1.186	1.030–1.366
Risk of MACE			
JKAP level at M1 after PCI (pg/mL)	0.045	0.950	0.904–0.999
Treg cells (%)	0.043	0.523	0.279–0.980
Scr (μmol/L)	0.016	1.036	1.007–1.066
hsCRP (mg/L)	0.026	1.317	1.034–1.678

All biochemical parameters were included in the regression analyses in ACS patients. OR, odds ratio; CI, confidence interval; SPPB, simple physical performance batter; JKAP, JNK pathway-associated phosphatase; Scr, serum creatinine; cTnI, cardiac troponin I; MACE, major adverse cardiovascular events; Treg, T regulatory; hsCRP, high-sensitive C-reactive protein.

**Figure 5:**
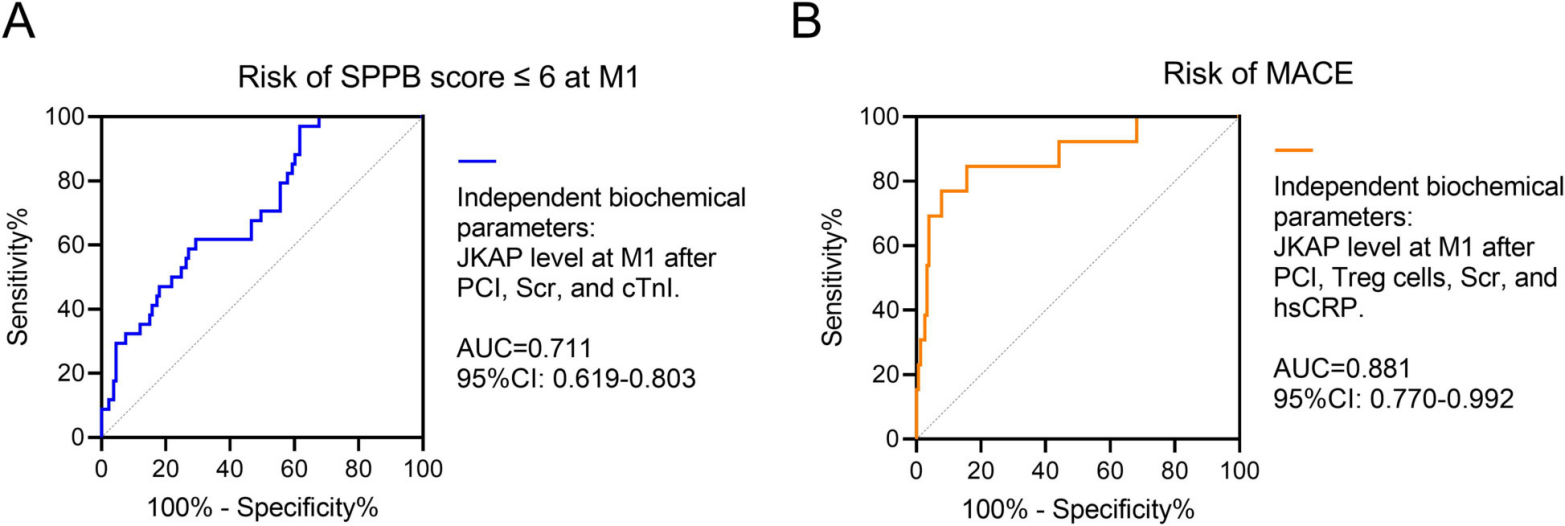
Independent biochemical parameters for prognosis. ROC analysis of independent biochemical parameters including JKAP levels at M1 after PCI, Scr, and cTnI for the risk of SPPB score ≤6 at M1 **(A)** ROC analysis of independent biochemical parameters including JKAP levels at M1 after PCI, Treg cells, Scr, and hsCRP for the risk of MACE **(B)**.

ROC curve analyses further revealed that independent biochemical parameters—including JKAP levels at M1 after PCI, Scr, and cTnI—had a good value (AUC = 0.711, 95% CI: 0.619–0.803) in predicting risk of an SPPB score of ≤6 at M1 in ACS patients who had undergone PCI (Figure 5A). Moreover, the independent biochemical parameters—including JKAP levels at M1 after PCI, Treg cells, Scr, and hsCRP—had an excellent value (AUC = 0.881, 95% CI: 0.770–0.992) in predicting risk of MACE in ACS patients who had undergone PCI (Figure 5B).

## 4 Discussion

### 4.1 General discussion

JKAP levels have been found to be dysregulated in a number of immunity- and inflammation-related diseases (22, 26–28). Serum JKAP levels are lower in patients with rheumatoid arthritis and are negatively correlated with systemic inflammation and general disease activity (26). JKAP is also lower in sepsis patients and is inversely associated with levels of TNF-α, IL-1β, and IL-17 and disease severity-related scales (27). Moreover, JKAP levels are lower in patients with chronic obstructive pulmonary disease; JKAP is also inversely related to pulmonary function and IFN-*γ* and IL-17A levels (28). Our earlier study had revealed that JKAP levels were lower in patients with coronary heart disease, which was negatively associated with CRP, inflammatory cytokines, and stenosis degree; even though the sample size of our previous study was relatively small, it implied the potential involvement of JKAP in ACS patients (22). The present study involved 173 ACS patients and found that JKAP levels were lower in ACS patients, which it was negatively correlated with blood glucose, hsCRP, cTnI, and a medical history of hyperlipidemia and diabetes. The reasons underlying these findings may include the following: (1) Atherosclerosis, aberrant immunity, and chronic inflammation are important triggers of ACS (19–21, 29). JKAP is able to repress the progression of atherosclerosis, immunity, and inflammation (9, 10, 16–18, 22). Through this connection, JKAP levels are reduced in ACS patients. (2) JKAP regulates the JNK, ERK, and NF-κB pathways (11, 12), which provide important signals for glycometabolism and lipid metabolism (30–32); thus, JKAP was negatively correlated with blood glucose and a medical history of hyperlipidemia and diabetes. (3) JKAP represses inflammation and atherosclerotic progression (16–18, 22); thus, JKAP was negatively correlated with hsCRP and cTnI. In addition, this study also found that JKAP levels were increased at M1 after PCI treatment compared to those before PCI possibly because the body is recovering after PCI, leading to revascularization and reduced inflammation; therefore, JKAP levels are higher following PCI.

JKAP is a regulator of CD4^+^ T-cell differentiation. In brief, it represses the CD4^+^ T cell from differentiating into Th1 and Th17 cells, and to some degrees promotes its differentiation into Th2 and Treg cells (18, 22, 33). Clinically, JKAP is negatively correlated with Th17 cells while it is positively associated with Th2 cells in patients with diabetes mellitus (34). JKAP is also positively correlated with Th1 cells and Th17 cells in patients with Alzheimer's disease (35). Moreover, a negative relation between JKAP and Th17 cells is also reported in patients with sepsis or chronic obstructive pulmonary disease (27, 28). Our previous study revealed that JKAP represses the CD4^+^ T cell from differentiating into Th1 and Th17 cells *in vitro* and *in vivo* and is negatively correlated with Th1 and Th17 cells in blood from patients with coronary heart disease (22). This present study found that JKAP was negatively associated with Th17 cells in ACS patients. The reasons underlying these findings may include the following: (1) JKAP represses Th17 differentiation by regulating ERK and NF-κB pathways (22). (2) JKAP plays an important role in regulating the TCR pathway (9, 16), and the TCR pathway is closely implicated in the Th17 differentiation (36).

Given its important role in regulating immunity and inflammation, JKAP has been reported to possess robust prognostic value across several diseases (27, 37, 38). For instance, a lower serum level of JKAP at admission is related to a higher risk of mortality in sepsis patients (27), and a lower level of JKAP at admission is also correlated with a higher risk of stroke recurrence and mortality risk in patients with acute ischemic stroke (37). Moreover, even though JKAP levels at admission are not related to outcomes in patients with rheumatoid arthritis, level after treatment are correlated with treatment response and remission to some degree in these patients (38). The present study found that JKAP levels before PCI (AUC = 0.721) and JKAP at M1 after PCI (AUC = 0.778) showed good values in predicting risk of MACE in ACS patients who had undergone PCI. Lower levels of JKAP were associated with a higher risk of MACE. The reasons for these finding may include the following: (1) JKAP represses systemic inflammation (16–18), and reduced inflammation contributes to a lower MACE risk in ACS patients (39). (2) JKAP attenuates the progression of atherosclerotic (22), and decreased atherosclerotic progression leads to a lower MACE risk in ACS patients (29). In addition, this present study also found that JKAP before PCI (AUC = 0.638) and JKAP at M1 after PCI (AUC = 0.649) could predict an SPPB score of ≤6 at M1 in ACS patients who had undergone PCI; however, the predictive values in identifying patients at risk of an SPPB score of ≤6 at M1 were not stronger compared to their predictive strength for MACE. This is possibly because SPPB is affected by numerous factors apart from cardiovascular condition recovery, such as age, original physical status, diet, and lifestyle.

The present study further analyzed the independent biochemical parameters involving JKAP associated with the risk of an SPPB score ≤6 at M1 and MACE. The findings demonstrated that JKAP levels at M1 after PCI proved to be an independent factor predicting lower risk of both an SPPB score ≤6 at M1 and MACE in ACS patients who had undergone PCI. Meanwhile, Tregs, Scr, hsCRP, and cTnI were also independent factors related to the risk of an SPPB score ≤6 at M1 or MACE. Significantly, the independent biochemical parameters comprising JKAP levels at M1 after PCI, Scr, and cTnI showed a robust value (AUC = 0.711) in predicting risk of an SPPB score ≤ 6 at M1; the independent biochemical parameters consisting of JKAP levels at M1 after PCI, Treg cells, Scr, and hsCRP also revealed an excellent value (AUC = 0.881) in predicting risk of MACE in ACS patients who had undergone PCI. These findings further confirm the prognostic value of JKAP in ACS patients who have undergone PCI.

### 4.2 Limitations

The limitations of the present study are as follows: First of all, serum JKAP levels were compared between ACS patients and healthy control subjects; however, there were no disease control subjects in this study. Therefore, the value of JAKP levels in the ability to distinguish between ACS and other cardiac/cardiovascular diseases can be explored in a future study. Second, the follow-up time in the present study was relatively short for comprehensive evaluation of MACE; thus, long-term evaluation can be explored in a future study. Third, the mechanism of JKAP in predicting MACE or physical function recovery was not directly investigated in this study, which can be explored in a future study. Fourth, blood samples were taken for JKAP level measurement in this study, while JKAP levels through other source samples were not used. Therefore, measurement of JKAP levels in other types of samples could be explored in a future study.

## 5 Conclusion

JKAP correlates with CD4^+^ T-cell subtypes and provides prognosis for ACS patients who have undergone PCI, potentially due to its role in immune modulation. However, further large-scale long-term follow-up studies are required for future validation.

## Data Availability

The original contributions presented in the study are included in the article/Supplementary Material, further inquiries can be directed to the corresponding author.
