# Supplementary material for: Serum JKAP as a potential prognostic biomarker in acute coronary syndrome patients undergoing percutaneous coronary intervention
Source: Front Cardiovasc Med. 2025 Dec 12;12:1631896. doi: 10.3389/fcvm.2025.1631896 (PMC12741847; doi:10.3389/fcvm.2025.1631896)
Supplement: Supplementary file 1 [file Image1.pdf]

Supplementary Materials

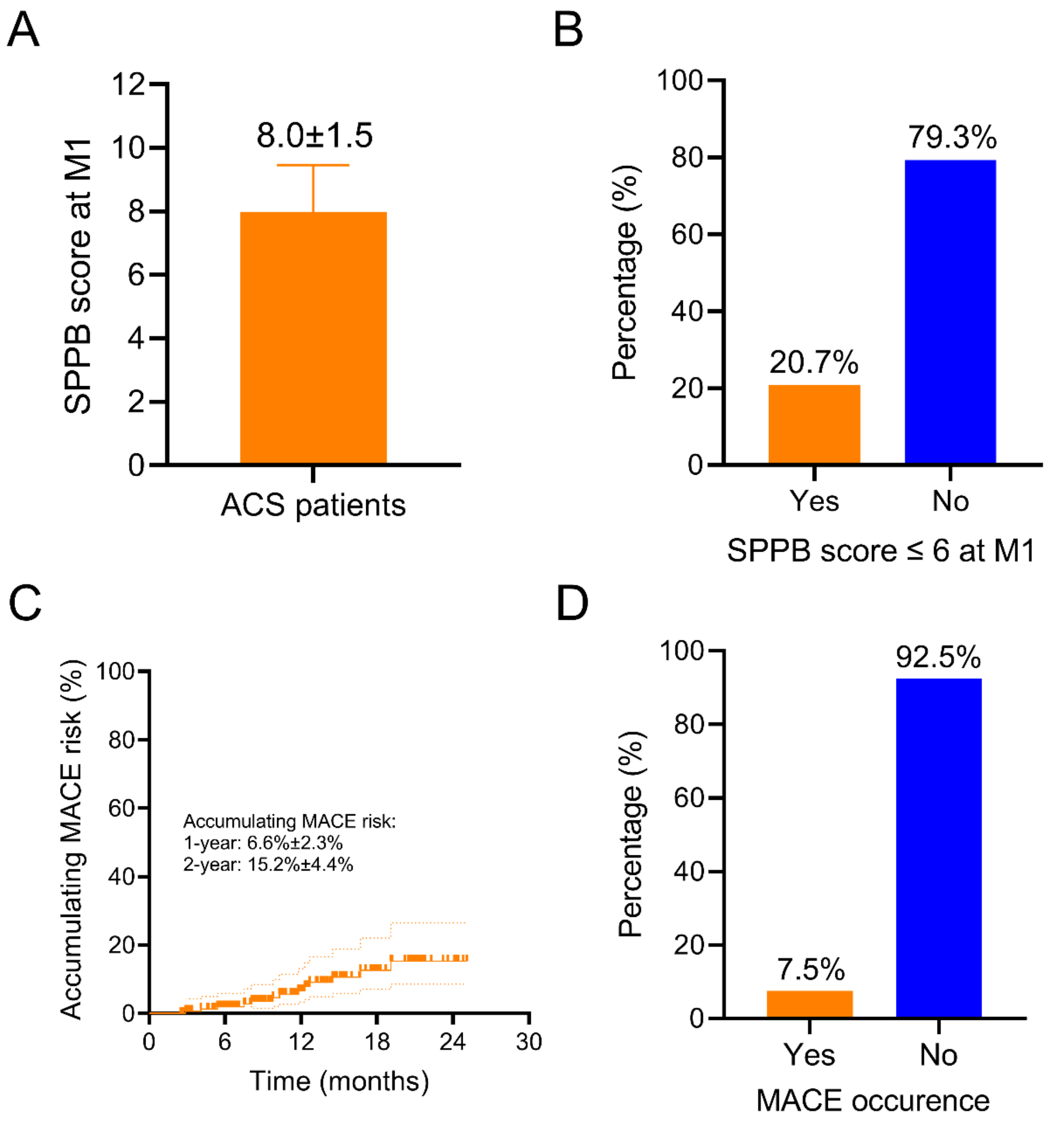

Supplementary Figure 1. Prognostic information. SPPB score at M1 (A), percentage of patients having SPPB score ≤6 at M1 (B), Kaplan-Meier curve revealing the accumulating MACE risk (C), percentage of patients occurred MACE (D).
